# Reliability of the Siemens Enzygnost and Novagnost Epstein–Barr Virus assays for routine laboratory diagnosis: agreement with clinical diagnosis and comparison with the Merifluor Epstein–Barr Virus immunofluorescence assay

**DOI:** 10.1186/1471-2334-13-260

**Published:** 2013-06-03

**Authors:** Christina Kreuzer, Klaus Udo Nabeck, H Roma Levy, Elisabeth Daghofer

**Affiliations:** 1 University of Graz, Institute for Hygiene, Universitätsplatz 4, Graz, 8010, Austria; 2 Siemens Healthcare Diagnostics Products GmbH, Emil-von-Behring-Str. 76, Marburg 35041, Germany; 3 Siemens Healthcare Diagnostics, 5210 Pacific Concourse Drive, Los Angeles, CA, USA

**Keywords:** EBV, Serodiagnosis, EIA, Microtiter, Enzygnost, Novagnost, Merifluor

## Abstract

**Background:**

Diagnosis of Epstein–Barr virus (EBV) infection is routinely conducted by clinical laboratories, especially to diagnose infectious mononucleosis. At an estimated general population incidence of 1:200, this represents a potentially significant testing burden. We evaluated the reliability of the Siemens Novagnost® and Enzygnost® EBV microtiter assays measuring VCA IgM and IgG, and EBNA-1 IgG for clinical diagnosis of EBV-related infectious mononucleosis.

**Methods:**

Remnant sera from 537 patients tested for EBV infection were used to compare the Siemens assays to each other and to the Merifluor assay. The Siemens assays are qualitative/semiquantitative, automatable enzyme immunoassays. The Merifluor assays are manual, qualitative indirect immunofluorescent assays. Testing was conducted on the Siemens and Merifluor assays in parallel. All assays were conducted and interpreted according to each manufacturer’s specifications. Agreement of serostatus between each of the three assays was assessed. Discrepant results were resolved using a third method (Mikrogen *recom*Line).

**Results:**

Final EBV serostatus indicated 2.9% of the population had an acute infection, 89.6% had a past infection, and 7.5% were EBV naive. All three assays demonstrated 100% agreement with acute infection. Agreement with past-infection serostatus was 99.1% for Enzygnost, between 86% and 98.8% for Novagnost, and 98.1% for Merifluor. Seronegative agreement was 100% for Enzygnost, 89.7% for Novagnost, and 92.3% for Merifluor.

**Conclusions:**

The Siemens Enzygnost and Novagnost EBV microtiter assays are suitable for clinical rule-in of acute EBV infection and for identifying EBV-naive individuals. Both assays also adequately identify remote EBV infections. Because these assays can be automated, they can improve speed and efficiency of EBV testing, especially in high-volume laboratories.

## Background

Epstein–Barr virus (EBV) is a ubiquitous pathogen endemic in most populations. According to the World Health Organization (WHO), approximately 95% of the adult population is infected, and in developed nations approximately 50% to 70% of exposures occur in adolescents or young adults. The majority of these infections are either asymptomatic or manifest with only minor upper respiratory symptoms, but approximately 30% of primary infections in adolescence or beyond present as infectious mononucleosis (IM). In the U.S., overall incidence of IM is estimated at 1:200, while incidence in 10- to 19-year-old adolescents is greater, at 6 to 8 cases per 1000 individuals per year [1-3].

Although EBV-related IM is typically a fairly benign disease, early and accurate diagnosis is valuable as it is highly communicable and can spread quickly in populations with a high density of young adults (such as among university students and military personnel). In rare instances however, sequelae of EBV-related IM can pose serious health risks, such as trauma-induced or spontaneous splenic rupture, fulminant helpatitis, or autoimmune hemolytic anemia. Furthermore, a great diversity of viral, bacterial, parasitic, and immunologic diseases can mimic IM symptoms, especially in early infection [4]. Thus, testing to confirm or rule out EBV infection has unquestioned diagnostic value and is standard clinical practice when classic symptoms (pharyngitis, fatique, fever, and adenopathy) have persisted for 2 or more weeks, especially in individuals between the ages of 10 and 30 years, and pregnant women [3]. Classically, the Paul–Bunnel heterophile agglutination test has been used for laboratory confirmation of acute EBV infection but several limitations exist. Sensitivity can be lower than desirable in early infection, and specificity when testing young children can be poor [3,5]. Specificity can also be affected by cross-reactivity with other viral and nonviral pathogens and some immunological markers [6]. Additionally, natural loss of heterophile antibodies impacts assessment of susceptibility or determination of other serostatus later in infection. Because heterophile agglutination assays must be conducted manually, they are not conducive to large-volume testing in midsize and large laboratories, where high throughput is required. Thus, specific serology to detect antibodies to viral proteins is used as both an adjunct and alternative to the heterophile test, and is most commonly directed at the antibodies made againsts viral capsid proteins (VCA) and Epstein–Barr nuclear antigens (EBNA).

Indirect immunofluorescence assays (IFA) using EBV-infected lymphocytes as the binding substrate are highly sensitive and specific for detecting serum antibodies to viral antigens. These assays can be used to diagnose active infections and differentiate acute from resolved infections and are frequently considered a gold standard, however close to 9% of results are uninterpretable [7]. IFA is a manual method which is more labor intensive than automated or semiautomated microtiter systems. It also requires substantial training of personnel for interpretation and is not easily standardizable; thus it too is not well suited for high-volume testing [8].

In contrast, serology for EBV-specific IgM and IgG antibodies by ELISA-type methods provides both high sensitivity and high specificity for acute infection [9,10], and may offer advantages for diagnostic confirmation earlier in the disease course—especially in those cases where symptoms are persistent but heterophile antibodies are not detectable. It can distinguish between acute and remote (past) infection, especially after heterophile antibodies have peaked or disappeared [6]. Furthermore, depending on the quality of the method, the serological profile can usually be determined reliably following a single blood draw. The ability to differentiate between susceptibility and acute vs. previous infection on the basis of a single blood draw is advantageous as it reduces the impact on both the patient and the laboratory. For the high-volume laboratory in particular, this advantage is further enhanced when semiautomated or automated assays are used to improve laboratory throughput.

Unfortunately, EBV serology is not always clear cut, as unusual serological patterns may be observed in up to 10% of patients [9,11]. While the presence of VCA IgM with or without VCA IgG typically signals acute infection, and the persistence of VCA IgG along with the appearance of IgG to the late protein EBNA-1 indicates past or resolving infection, unusual serological patterns such as isolated EBNA-1 or isolated VCA IgG can confound interpretation. Thus, to determine their usefulness for regular diagnostic laboratory testing, we evaluated the serological profiles interpreted from the combined results of the Siemens Novagnost VCA IgM, VCA IgG, and EBNA-1 IgG assays, and the combined results of the Enzygnost Anti-EBV/IgG and Anti-EBV/IgM II microtiter assays to rule in, or rule out, acute EBV infection. Our goal was to show that serostatus assignment could be achieved on the basis of a single blood sample by comparing the agreement of the results generated by the Siemens assays with the final empirically derived EBV serostatus for each sample.

## Methods

The serological assignments derived from combined analysis of the three Novagnost EBV enzyme immunoassays (VCA IgM, VCA IgG, and EBNA-1 IgG) were compared to the serological assignments derived from combined analysis of the two Enzygnost enzyme immunoassays (Anti-EBV IgG and Anti-EBV/IgM II). The Novagnost and Enzygnost assays are products of Siemens Healthcare Diagnostics GmbH (Marburg, Germany). Serological assignments were also made using the combined analysis of the two Merifluor EBV immunofluorescence assays (IgG and IgM: Meridian Bioscience Europe, Nivelles, Belgium). In total, five Siemens assays and two Merifluor assays were used in this study.

Samples were analyzed in parallel. A final serostatus (negative, acute, or past infection) was assigned to each sample on the basis of the combined results of all three assay sets, as defined by agreement between at least two of the three test types. For example, to be serostatus-negative, both IgM and IgG results had to be negative according to the assignments determined by at least two of the three methods. If results were indeterminate, a fourth assay (*recom*Line EBV IgG and IgM line immunoassays; Mikrogen Diagnostik, Neuried, Germany) was used as a referee method to designate serostatus. Following serostatus assignment for each sample, agreement between the assigned status and the individual results from the Enzygnost, Novagnost, and Merifluor methods were compared to determine their ability to correctly differentiate between EBV-naive status, acute infection, and past infection on the basis of a single blood draw.

### Assay design

The Novagnost EBV VCA IgG, Novagnost EBV VCA IgM, and Novagnost EBV EBNA-1 IgG assays are qualitative/semiquantitative enzyme immunoassays (ELISAs) conducted in microtiter strip wells. In the VCA IgG and IgM assays, wells are coated with recombinant p18 antigen; in the EBNA assay, wells are coated with recombinant EBNA-1 antigen. In each assay, EBV antibodies in patient samples create antibody–antigen complexes, after which they are incubated with peroxidase-labeled anti–human IgG (VCA IgG and EBNA-1 IgG assays) or IgM (VCA IgM assay). Following subsequent washing steps, samples are incubated further with tetramethylbenzidine (TMB). After addition of a stop solution, color intensity is measured at 450 nm. Absorbance, which is proportional to the antibody level in the patient sample, may be calculated manually, although in this study, it was determined automatically using the Siemens BEP® III analyzer. The level of EBV antibodies in the sample is provided in Novagnost units (U) for all three assays. According to the manufacturer, results <8.5 U should be considered negative and those >11.5 U, positive; while those between 8.5 and 11.5 U fall within the gray zone. For the purposes of this study, however, results >11.5 U were considered positive, while all results ≤ 11.5 U were considered negative.

Both the Enzygnost Anti-EBV/IgG and Enzygnost Anti-EBV/IgM II assays are qualitative/semiquantitative enzyme immunoassays (ELISAs) conducted in microtiter strip wells coated with EBV antigens originally derived from EBV-infected lymphoblastoid cells undergoing active viral replication. For each assay, control wells contain antigen from EBV-infected lymphoblastoid cells in which viral synthesis has been blocked. Reactivity is defined by the difference in absorbance (∆A) measured at 450 nm between the sample and control antigen wells. If results are calculated manually, a value correction factor (CF) must be applied; in our study, however, values were determined using the BEP III analyzer, which automatically includes the CF in its results calculation. As with the Novagnost assays, the Enzygnost assays employ an indirect EIA format; color intensity increases with higher serum concentrations of EBV antibody. For the IgM assay, ∆A ≥0.120 is considered positive, while ∆A <0.120 is negative. For the IgG assay, ∆A of 0.200 marks the upper retest limit, while for negative samples, ∆A of <0.100 is the cutoff value. Results are equivocal if 0.100 ≤ ∆A ≤ 0.200.

The Merifluor EBV VCA IgM and EBV VCA IgG IFA methods are manual, qualitative indirect immunofluorescent assays. EBV-infected lymphocytes from Burkitt lymphoma tumors are incubated with patient serum and coated onto slides. After washing, cells complexed with bound anti-VCA antibodies are incubated with either anti–human IgM or anti–human IgG tagged with fluorescein. Infected cells glow light yellow to green using fluorescence microscopy if the patient sample is positive for the antibody being investigated. The sample is considered to be positive if approximately 10% to 20% of the cells in each field fluoresce upon visual inspection.

The Mikrogen assays use recombinant proteins developed from the antigenic components of early antigen (EA: p54, p138), immediate early antigen (IEA: BZLF-1, ZEBRA), VCA (p18, p23), and EBNA-1 (p72). Antigens are bound to nitrocellulose test strips. The IgM test strip is coated with p23, ZEBRA, p138, and p54 antigens, and the IgG test strip is coated with EBNA-1, p18, p23, BZLF-1, p138, and p54 antigens. Strips blotted with each antigen are cut into smaller strips, each of which is incubated with patient serum. After washing, incubation continues with anti–human IgG or anti–human IgM coupled with horseradish peroxidase. Upon addition of chromogen, a positive reaction is noted by a dark band appearing on the test paper. The Mikrogen assays are conducted and read manually.

### Patient population and assignment of serostatus

Samples were derived from remnant sera drawn at our laboratory (Institute of Hygiene, University of Graz) from 537 patients who originally had been referred for testing after clinical evaluation and history suggested possible EBV infection or exposure. Blood samples were collected sequentially as patients were referred. Because this study was non-interventional and the sample sera were originally obtained in the course of regular clinical care and not for experimental purposes, this study required neither patient consent nor ethics board review per Austrian regulations as outlined in the document “Scientific Guidance for the Conduct of Non-interventional Studes (NIS) in Austria”. This guidance document is issued by the Bundesamt für Sicherheit im Gesundheitswesen (Federal Office for Health Service Security), and can be accessed at http://www.basg.gv.at/uploads/tx_basginfobox/L_Z61_Guidance_NIS_AT.pdf.

### Statistical analysis

Although immunofluorescent methods are highly sensitive, such tests, including the Merifluor test, are not considered to be definitive reference methods. All tests in this study were therefore evaluated in terms of statistical agreement according to the methods outlined in the FDA guidance document on appropriate statistical methods used for evaluating diagnostic tests [12]. Analysis was conducted by the investigators with the input of two biostatisticians.

## Results

A total of 537 sera were available for testing using the Siemens and Merifluor methods. Samples were assigned a final interpretation of negative, acute infection, past infection, or indeterminate on the basis of agreement between results of the three assays used. Status was assigned if results of at least two of the three methods (Novagnost, Enzygnost, Merifluor) yielded the same interpretation. If at least two of the three methods for any serum sample did not agree or were deemed indeterminate, the Mikrogen assay was used to resolve EBV status. If no resolution could be achieved using the Mikrogen assay, the sample was excluded from the study. Interpretation algorithms for the Siemens, Merifluor, and Mikrogen assays are presented in Tables 1, 2, and 3.

**Table 1:** Serological assay interpretation per the Enzygnost and Merifluor assays

**anti-EBV IgM**	**anti-EBV IgG**	**Patient status**
–	–	negative^a^
+	–	Primary infection
+	+	Primary infection
–	+	Past infection

^a^Retest in 7 days if suspicion remains high.

**Table 2:** Serological assay interpretation per the Novagnost assay

**VCA IgM**	**VCA IgG**	**EBNA-1**	**Patient status**	**Alternative interpretation**
–	–	–	Probably not infected^a^	
+	–	–	Primary early phase infection	● Possible cross-reactivity with primary CMV infection IgM
+	+	–	Primary acute infection	
–	+	+	Past infection	
–	+	–	Uncertain result	● Primary infection without VCA IgM
				● Past infection with EBNA loss
–	–	+	Uncertain result	Very rare profile of past infection:
				● Possible loss of VCA IgG
				● Possible EBNA false positive
+	+	+	Uncertain result	● Just past primary infection
				● Viral reactivation
				● Cross-reaction with CMV primary infection IgM
				● Nonspecific polyclonal IgM stimulation from a previous cross-reacting
				● infectious agent

^a^ Retest in 7 days.

**Table 3:** Serological assay interpretation per the Mikrogen assay

**IgG strip**					**IgM strip**				**IgA**	**Patient status**
EBNA-1	p18	p23	BZLF1	EAs (p54, p138)	p54	p138	ZEBRA	p23		
–	–	–	–	–	–	–	–	–	–	EBV negative
–	–	+ or –	+ or –	+ or –	+	+ or –	+ or –	+ or –		Primary infection
–	–	+ or –	+ or –	+ or –	+ or –	+	+ or –	+ or –		
–	–	+	+	weak + or -	–	–	–	–		Past infection
–	–	+	+	weak +	weak +	weak +	weak +	weak +		Secondary reactivation^a^
+^b^	+	+	+	+	– or weak +	– or weak +	– or weak +	– or weak +	EA + BZLF1+ VCA+	Reactivation
–	–	+	+	weak +	weak +	weak +	weak +	weak +	High	Nasopharyngeal cancer
										EBV-associated lymphoma
IgG/IgM/IgA: one isolated band (with the exception of the markers IgG EBNA-1 and IgG VCA) in only one antibody class and if all other antibody classes are negative										No definitive test result (retest after 2–3 weeks)

^a^ No clinical relevance.

^b^ In a very few cases, loss of anti–EBNA-1 antibodies.

Of the available samples, results from only 518 were ultimately included in this study. Nineteen sera could not be used: 11 samples lacked sufficient volume for testing by all systems, and status could not be resolved for the remaining 8 samples. Of the 518 analyzable samples, the same 15 sera were IgM^+^ according to all three assays, indicating the presence of an acute infection. Two of the 15 IgM^+^ sera were also IgG^–^ according to the Merifluor assay, while 1 was IgG^–^ and 3 were borderline IgG^+^ according to the Enzygnost assay and 3 were IgG^–^ according to the Novagnost assay. All of these 15 IgM^+^ assays were also EBNA-1^–^, confirming their acute infection serostatus. Table 4 shows the final assigned EBV serostatus for all 518 samples. Overall results for each assay type were compared to the assigned EBV status and are displayed in Tables 5, 6, and 7. A synopsis of assay agreement with EBV status is presented in Table 8.

**Table 4:** Final EBV status for all 518 analyzable samples

	**Number**	**Percent of population**
Negative	39	7.5
Acute infection	15	2.9
Past infection	464	89.6

**Table 5:** Results using the Enzygnost assays as compared to established EBV status

			**Enzygnost interpretation**			
		**Negative**	**Acute infection**	**Past infection**	**Indeterminate**	**% agreement with EBV status**
EBV status	Negative (N = 39)	39	0	0	0	100
	Acute infection (N = 15)	0	15	0	0	100
	Past infection (N = 464)	2	2	460	0	99.1

**Table 6:** Results using the Novagnost assays, including indeterminates, as compared to established EBV status

		**Novagnost interpretation**						
		**Determined**			**Indeterminate**			
		**Negative**	**Acute infection**	**Past infection**	**Isolated EBNA-1 (probable past infection)**	**Isolated VCA IgG (EBNA-1 lost, probable past infection)**	**All positive (probable past infection)**	**% agreement with EBV status**
	Negative^a^ (N = 39)	35	1	1	0	0	0	89.7
	Acute infection (N = 15)	0	15	0	0	0	0	100%
	Past infection (N = 464)	5	0	399	8	55	2	86^b^
EBV status								98.8^c^
								87.7^d^
								97.8^e^
								99.6^f^

^a^ Determination could not be made for 2 sera which did not match any of the indeterminate types listed in the table.

^b^ Agreement if all indeterminate results are included in the analysis.

^c^ Agreement if all indeterminate results are excluded from the analysis (i.e., 399/404).

^d^ Agreement if all isolated EBNA-1 results are assumed to represent past infections.

^e^ Agreement if results are considered indeterminate because they are VCA IgG^+^–only (isolated VCA IgG) are instead considered positive, and thus also most likely represent past infections.

^f^ Agreement if all isolated EBNA-1 and all isolated VCA IgG^+^ results are assumed to represent past infections.

**Table 7:** Results using the Merifluor assays as compared to established EBV status

		**Merifluor interpretation**				
		**Negative**	**Acute infection**	**Past infection**	**Indeterminate**	**% agreement with EBV status**
EBV status	Negative (N = 39)	36	1	2	0	92.3
	Acute infection (N = 15)	0	15	0	0	100
	Past infection (N = 464)	1	5	455	3	98.1

**Table 8:** Overall agreement of sample results for the Enzygnost, Novagnost, and Merifluor assays with the established EBV status, including 95% confidence intervals (95% CI)

	**% agreement with established EBV status**		
	**Negative (N = 39)**	**Acute infection (N = 15)**	**Past infection (N = 464)**
	**[95% CI]**	**[95% CI]**	**[95% CI]**
Enzygnost	100	100	99.1
	[90.97% – 100%]	[78.2% – 100%]	[97.81% – 99.75%]
Novagnost	89.7	100	86^a^, 98.8^b^
	[75.78% – 97.13%]	[78.2% – 100%]	[82.5% – 89.02%]^a^, [97.1% to 99.6%]^b^
Merifluor IFA	92.3	100	98.1
	[79.13% – 98.38%]	[78.2% – 100%]	[96.35% – 99.11%]

^a^ Agreement if all indeterminate results are included in the analysis.

^b^ Agreement if all indeterminate results are excluded from the analysis.

### Enzygnost assay performance

In general, the Enzygnost assay results demonstrated good total agreement with each of the three established EBV status types: 100% for EBV-naive patients (95% CI: 90.97% to 100%), 100% for acute infection (95% CI: 78.2% to 100%), and 99.1% for past infection (95% CI: 97.81% to 99.75%).

### Novagnost assay performance

Overall, Novagnost agreement with the established EBV status for EBV-naive sera (89.7%, 95% CI = 75.78% to 97.13%) was somewhat lower than agreement using the Enzygnost assays, but it was equally as good at detecting acute infections (100%, 95% CI: 78.2% to 100%).

Interpreting remote (past) infection using the Novagnost assay can be more challenging. Unlike the Enzygnost and Merifluor assays which do not differentiate between antibody types generated against total EBV antigens, Novagnost specifically detects antibodies to either VCA or EBNA-1 (depending on the assay), and thus has the potential to report atypical/rare serotypes. Additionally, approximately 5% of the population never produces antibodies to EBNA-1 [7]. Thus, it could be expected that approximately 5% of the group diagnosed with acute infection were actually remotely infected, which could affect the agreement for EBV acute infection as well as past-infection status. This proved not to be the case, however, as all patients in our study population with the EBNA-1–lost serotype (isolated VCA IgG^+^) were VCA IgM^–^, and thus were determined to have experienced a past infection (although they are reported in Table 6 as indeterminates). We analyzed the results from the past-infection study population by both including and excluding atypical, i.e., indeterminate, serotypes. When all indeterminate serotypes were excluded, 98.8% of samples correctly reported a remote infection (95% CI: 97.1% to 99.6%). If all serotypes were included, agreement with the established EBV status was 86% (95% CI: 82.5% to 89.02%). If only the samples that were isolated–VCA IgG^+^ were presumed to reflect past infections in which EBNA-1 IgG had been lost, however (see discussion below), then agreement with the established EBV past-infection status rises to 97.6%; and if both isolated–EBNA-1^+^ and isolated–VCA IgG^+^ serotypes are considered to reflect past infection, agreement was as high as 99.6%.

### Comparison to Merifluor assay performance

The Enzygnost and Novagnost assays were found to detect acute infection, past infection, and EBV-naive status at least as well as the Merifluor assays. The Merifluor assay agreement for seronegativity (92.3%, 95% CI: 79.13% to 98.28%) was somewhat lower than that of the Enzygnost assay (100%), but agreement for detecting acute infection was identical for the Merifluor and Enzygnost assays (100%, 95% CI: 78.2% to 100%). Merifluor agreement to past infections was 98.1% (95% CI: 96.35% to 99.11%), which was slightly lower than agreement displayed by the Enzygnost assay, but greater than agreement displayed by the Novagnost assay when all serotypes were considered in the analysis. However, Merifluor agreement to past infections was slightly lower than the Novagnost assay past infection–agreement if indeterminate serotypes were excluded from the Novagnost analysis.

## Discussion

We undertook this study to evaluate efficacy of the Siemens Novagnost and Enzygnost EBV assays in routine clinical use as an aid to determining EBV exposure and serostatus. The samples were drawn as part of normal clinical practice from patients presenting with symptoms suspicious for EBV infection, or who had been exposed to a person with a known EBV active infection. In our estimation this study group was reasonably representative of populations in industrialized nations at normal risk for EBV infection [3], as 89.6% of our patient results demonstrated previous exposure, 7.5% of the patients were determined to be EBV naive, and only 2.9% were ultimately diagnosed with an acute EBV infection.

For EBV serology to be a practical aid in the diagnosis of IM (its most common clinical usage), it should either rule in or rule out an acute infection with a high degree of accuracy following a single blood draw. Additionally, it should also accurately distinguish primary acute infection from past infection, as well as identify those individuals who are EBV naive, and who therefore remain at risk of contracting an EBV infection. Ideally, results should not be confounded by unusual serological profiles that require additional testing to achieve resolution (e.g., isolated VCA IgG, i.e., VCA IgM^–^/VCA IgG^+^/EBNA IgG^–^; isolated EBNA, i.e., VCA IgM^–^/VCA IgG^–^/EBNA IgG^+^; or all markers positive, i.e., VCA IgM^+^/VCA IgG^+^/EBNA IgG^+^). Assays such as Enzygnost and Merifluor, which report only qualitative results for total viral IgM and IgG, are less subject to such confusion. Results from these two assays demonstrated the greatest concordance with EBV-naive status, and thus would be expected to perform reliably for ruling out EBV infection. The Novagnost assay also demonstrated good agreement with EBV-negative status, although concordance was not as good as that achieved by either the Enzygnost or Merifluor assays. This reduced concordance is directly attributable to the larger number of samples that were either incorrectly assigned or indeterminate (4 for Novagnost, versus 0 for Enzygnost and 3 for Merifluor). It is possible that we would have been able to resolve the 4 indeterminate samples had we retested these individuals 2 to 4 weeks later, following the manufacturer’s guidelines; this resolution might possibly have improved the concordance for the Novagnost assay. However, since one of the stated purposes of our study was to determine serostatus on the basis of a single blood draw, only, we elected to not conduct later testing. It is important to note, however, that the manufacturer clearly states that gray zones exist for the Novagnost assays (between 8.0 and 11.5 U) and the Enzygnost IgG assay (between 0.1 and 0.2 U), and recommends retesting patients with sera yielding gray zone values two to four weeks after the original blood draw. If samples continue to report in the gray zone, they should be considered negative. Clinical laboratories should keep this in mind and consider retesting patients accordingly if alternative methods are not available for resolving indeterminate samples.

All three of the assays demonstrated excellent and identical concordance (100%) with the final EBV status for sera from patients with an acute primary infection: all three correctly identified those sera that were either IgM^+^ only, or were IgM^+^ in the presence of either total IgG or specific VCA IgG. Although it is not possible to tell if the Enzygnost and Merifluor assays are detecting EBNA-1 IgG as part of the IgG population, the absence of EBNA-1 IgG detection by the Novagnost assay for all 15 acute sera confirms that none of the assays are detecting EBNA-1 in these samples. This suggests that both of the Siemens assays can be considered reliable for detecting a primary acute EBV infection on the basis of a single blood draw. Later retesting to establish a positive diagnosis of infectious mononucleosis is not likely to be necessary unless phlebotomy has occurred so early in the disease process that the patient has not yet developed a sufficient antibody response.

The greatest challenge in EBV testing can lie in differentiating between an acute infection and a past infection, with or without reactivation. The Enzygnost assay performed at least as well as the Merifluor assay in this respect. The Enzygnost assay has also been shown to perform well in other studies [13,14], although Bruu et al. noted that because separate EBNA-1 detection is lacking, it is not possible to determine if IgM positivity is a true indication of primary acute infection, or if it might represent reactivation in those cases where EBNA-1 has been lost or was never generated [8,15]. Since reactivation is typically thought of as a rare event, however, this is likely of little consequence for diagnosis in the general population.

Atypical serotypes may be observed when using assays such as Novagnost, as they detect EBNA-1 IgG specifically, in addition to IgG to VCA; thus, their interpretation can constitute a diagnostic challenge [9,16]. Garcia et al. determined that the isolated EBNA-1 serotype occurred in up to 50% of their patients ultimately diagnosed with a latent EBV infection, and further noted that knowledge of latent infection is especially critical preoperatively in prospective solid organ transplant patients [17]. In a study by Nystad et al., the simultaneous detection of VCA IgM, VCA IgG and EBNA-1 was associated with primary infection in 42% of their patients, although in at least 23% of the study group it was associated with clinically inconsequential EBV reactivation [18]. In contrast, Klutts et al. found that 77.8% of patients who were VCA IgM^+^/VCA IgG^+^/EBNA-1^+^ proved to have had a past infection, were recovering from a primary infection, or were manifesting EBV reactivation [9]. Debyser et al. also associated the triple-positive serotype with reactivation, noting that reactivation can also be indicated by the VCA IgM^+^/VCA IgG^–^/EBNA-1^+^ serotype, but proposed that reactivation is only relevant in patients suffering from (or as in the case with Garcia et al., [17] subject to) immune suppression. Additionally, they suggested that isolated VCA IgG, especially when the IgG titer is high, was indicative of persistent or chronic EBV infection [19]; however, 69.6% of patients with the VCA IgG^+^ only serotype in the study by Klutts et al. [9] were diagnosed with a past infection.

In our opinion, the isolated VCA IgG^+^ results generated by the Novagnost assay are the most problematic when screening otherwise presumed healthy individuals. Although, as Klutts suggests, the majority of these likely represent remote infections, it is not possible to tell from the assay results alone if some of these sera might be indicative of a late active infection with early IgM decline and weak EBNA response, or a remote infection in which EBNA IgG has been lost or never generated. Since there is the possibility that individuals with isolated VCA IgG^+^ could have an acute infection, sera such as these should be tested for VCA IgG avidity or their status should be confirmed by some other method, such as Western blot or PCR [20-22]. Since the goal of our study was to evaluate only the performance of the microtiter assays as they compared to each other, we did not conduct any further evaluation of any of the indeterminate sera. While this might be considered a limitation of the study, we felt that further testing was not warranted as the original testing indicated 100% concordance between the three methods for detecting acute infection, and in all likelihood, the indeterminate sera truly represented remote infections.

Despite the difficulties that EBNA IgG detection can cause in interpreting rare serotypes, it is still considered to be a valuable component of EBV testing; absence of EBNA IgG in the presence of VCA IgM is considered definitive for ruling in acute infection, while the presence of EBNA IgG typically rules out acute infection, even if VCA IgM antibodies persist. Although, as shown by Nystad and Klutts in the above discussion, EBNA-1 can be indicative of a resolving infection. VCA IgG positivity, however, can be equally attributable to acute, resolving, or remote infections. Furthermore, VCA IgG levels may fall to undetectable levels in some individuals following acute infection, while EBNA IgG generally persists for life [23].

In our hands, the Novagnost assay performed about as well as both the Enzygnost and Merifluor assays for detecting active primary infection, but because the Novagnost assay reports the presence of antibodies to EBNA-1 in addition to the VCA antibodies, it was more difficult to appropriately classify all past infections. Of the total 464 sera determined to represent past infections, 65 expressed atypical serotypes and were subsequently classified as indeterminate. However, the 55 samples demonstrating isolated VCA IgG^+^ status constitute approximately 12% of the study population, which is consistent with the expected percentage of individuals who either never develop EBNA-1 antibodies or in whom antibody production is lost. Since VCA IgM rarely persists beyond acute infection, it is likely that these 55 samples do, indeed, represent patients with remote exposure to EBV, although it cannot be determined if these sera represent reactivation or chronic infection. Additionally, eight samples were positive only for EBNA-1 IgG, while two samples were positive for all markers; either of these profiles could be interpreted as past infection as shown in Table 2, and by the arguments presented by other researchers cited in the previous paragraphs.

## Conclusion

Overall, we found that both the Siemens Enzygnost and Novagnost EBV assays demonstrated very good agreement with assigned serostatus. Additionally, the ease with which these two assays may be used makes them well suited for laboratory assessment of infectious mononucleosis caused by primary EBV infection. Both assays can be automated and generate the same quality of results as manual assays, but require minimal hands-on involvement; this is a clear advantage in a busy, high-volume laboratory. Furthermore, because the Enzygnost assay requires reagents for detection of only two antibodies, it offers a considerable cost savings. Despite the potential for indeterminate results using the Novagnost assay, we found the test of practical use, especially considering the time-saving convenience of its ready-to-use reagents and their long stability.

## Abbreviations

(EBV): Epstein–Barr virus; (EA): Early antigen; (IEA): Immediate early antigen; (VCA): Viral capsid antigen; (EBNA): Epstein–Barr nuclear antigen.

## Competing interests

KUN and HRL are employees of Siemens Healthcare Diagnostics. Reagents and assay test kits were provided by Siemens. Neither ED nor CK received any financial support from Siemens. None of the authors have any other financial or nonfinancial competing interests.

## Authors’ contributions

KUN — Study coordinator: Initiated the study, helped develop the protocol, provided all reagents, supported and monitored the study site, analyzed the data, wrote the initial clinical trial report, provided critical review of the final manuscript. CK — Selected patient samples, conducted testing, interpreted results, and provided critical review of the final manuscript. ED — Head of laboratory: Designed the protocol; selected patient samples; oversaw all facets of the study, testing, and data analysis; and provided critical review of the final manuscript. HRL — Analyzed data, reviewed applicable literature, provided additional interpretation of results in accordance with other studies/literature, wrote the final manuscript. All authors read and approved the final manuscript.

## Pre-publication history

The pre-publication history for this paper can be accessed here:

http://www.biomedcentral.com/1471-2334/13/260/prepub
